# Does the liposuction method influence the phenotypic characteristic of human adipose-derived stem cells?

**DOI:** 10.1042/BSR20150067

**Published:** 2015-06-22

**Authors:** Anna Bajek, Natalia Gurtowska, Lidia Gackowska, Izabela Kubiszewska, Magdalena Bodnar, Andrzej Marszałek, Rafał Januszewski, Jacek Michalkiewicz, Tomasz Drewa

**Affiliations:** * Department of Tissue Engineering, Nicolaus Copernicus University, 85–092 Bydgoszcz, Poland; † Chair of Immunology, Nicolaus Copernicus University, 85-094 Bydgoszcz, Poland; ‡ Department of Clinical Pathomorphology, Nicolaus Copernicus University, 85-094 Bydgoszcz, Poland; § Department of Microbiology and Immunology, The Children's Memorial Health Institute, 04-730 Warsaw, Poland; ║ Department of Urology, Nicolaus Copernicus Hospital, 87-100 Torun, Poland

**Keywords:** adipose-derived stem cells, liposuction, markers' extensive analysis, phenotypic characterization

## Abstract

Adipose-derived stem cells (ASCs) possess a high differentiation and proliferation potential. However, the phenotypic characterization of ASCs is still difficult. Until now, there is no extensive analysis of ASCs markers depending on different liposuction methods. Therefore, the aim of the present study was to analyse 242 surface markers and determine the differences in the phenotypic pattern between ASCs obtained during mechanical and ultrasound-assisted liposuction. ASCs were isolated from healthy donors, due to mechanical and ultrasound-assisted liposuction and cultured in standard medium to the second passage. Differentiation potential and markers expression was evaluated to confirm the mesenchymal nature of cells. Then, the BD LyoplateTM Human Cell Surface Marker Screening Panel was used. Results shown that both population of ASCs are characterized by high expression of markers specific for ASCs: cluster of differentiation (CD)9, CD10, CD34, CD44, CD49d, CD54, CD55, CD59, CD71 and low expression of CD11a, CD11c and CD144. Moreover, we have noticed significant differences in antigen expression in 58 markers from the 242 studied. Presented study shows for the first time that different liposuction methods are not a significant factor which can influence the expression of human ASCs surface markers.

## Abstract (precis)

Statistical analysis revealed significant differences in antigen expression of 58 markers of the 242 studied. The method of liposuction has no significant impact on antigens profile in cultured ASCs (adipose-derived stem cells).

## INTRODUCTION

The first application of tissue engineering was based on the use of differentiated cells of the adult organism [1]. However, isolation of such cells was associated with a high risk of invasive and transplantation of pathologically changed cells [2]. Over the years, the range of available cell populations for tissue engineering has increased significantly. The unique properties of stem cells make them a good candidate for tissue engineering [3].

Since the identification of stem cells in bone marrow (BMSCs) 40 years ago, they have become a standard tool of regenerative medicine, mainly due to high differentiation potential [4,5]. However, the bone marrow aspiration is painful and requires general anaesthesia, furthermore the yield of isolated cells is usually low [5]. An alternative source of stem cells is the adipose tissue, from which large quantities of cells are isolated during a much safer and less time consuming liposuction procedures [6]. Furthermore, the high incidence of cells in the adipose tissue reduces the need for long-term culture *in vitro* and diminishes the risk of chromosomal abnormalities. Nevertheless, a characterization of adipose-derived stem cells (ASCs) population remains unclear. The sample collection for ASCs isolation raises a lot of controversy. Researchers use the adipose tissue, which is a waste product of abdominoplasty treatments, cosmetic surgery or liposuction [7]. Liposuction is currently the most commonly performed aesthetic surgery in the world. It is well-tolerated, safe and less invasive than bone marrow aspiration, being cheaper at the same time. However, there are different methods of liposuction. All previous attempts to determine the exact phenotype of ASCs and identify specific markers for these cells failed. There also is no comprehensive analysis of ASCs obtained during different liposuction methods. Therefore, the aim of the present study was the analysis of 242 surface markers and determining the difference in phenotypic patterns between ASCs obtained during mechanical and ultrasound-assisted liposuction.

## MATERIALS AND METHODS

### Tissue sampling

The Local Bioethical Committee of Nicolaus Copernicus University approved all procedures (no. KB 287/2011).

Human subcutaneous adipose tissue samples were obtained as waste material after mechanical- (MAL) and ultrasound-assisted liposuction (UAL; from the abdomen or hip region) upon informed consent. All patients, in both procedures, were selected according to similar body mass index (BMI) values (24.05±2.3) and age (37±1.4).

### Isolation and culture of adipose-derived stem cells

Adipose tissue, obtained from both procedures, was weighed and extensively washed with sterile PBS containing a 1% solution of penicillin/streptomycin and amphotericin B (Sigma) to remove blood cells, saline and anaesthetics. Resected material was digested enzymatically with 0.075% collagenase type I (Sigma) in Dulbecco's modified Eagle's medium (DMEM), for 30 min at 37°C with intermittent shaking. After incubation time, an equal volume of DMEM/Ham's F-12 medium (PAA) supplemented with FBS (Sigma) was added and neutralized the enzyme activity. The digested adipose tissue was centrifuged for 5 min at 200 ***g***. The cell-containing pellet was resuspended in DMEM/Ham's F12 and passed through 100 μm of cell strainer (BD Bioscience) due to removing debris and obtaining the SFV (stromal-vascular fraction) containing the ASCs. After centrifugation, the pellet was resuspended in DMEM/Ham's F-12 supplemented with 10% FBS, 1% amphotericin B and 1% penicillin/streptomycin. Single cell suspension was seeded according to the rule: 15ml of lipoaspirate for 25cm^2^ of culture dish. The primary cultures were established and maintained in 5% CO_2_ incubator at 37°C in a humidified atmosphere. The medium was changed every second day until cells reached 80%–90% of confluence.

### Characterization of ASCs

Cells, after second passage, were analysed for their ability to differentiate into the adipogenic, osteogenic and chondrogenic lineage. Differentiation into adipocytes and osteocytes was performed in a monolayer. Cells were plated at a density of 5 × 10^4^/well and 3 × 10^4^/well respectively and cultured at 37°C for 2 weeks in differentiation medium. Adipogenic differentiation was induced by DMEM/Ham ‘ s F12 (3:1) supplemented with 10% FBS, 10 μm of insulin (Sigma), 1 μM dexamethasone (Sigma), 5 μg/ml amphotericin B and 100 μg/ml penicillin/streptomycin. Differentiation process was visualized by staining with the use of red oil. Osteogenic differentiation was induced by DMEM/Ham ’ s F12 (3:1) supplemented with 15% FBS, 50 mM ascorbic acid (Sigma), 0.1 mM dexamethasone (Sigma), 5 μg/ml amphotericin B and 100 μg/ml penicillin/streptomycin. Differentiated ASCs were evaluated by calcified extracellular matrix (ECM) deposition using von Kossa assay (Bio-Optica).

Induction of chondrogenic differentiation was carried out in 3D pellet culture. For 3D culture, ASCs were resuspended at a density of 1 × 10^6^ in 0.5 ml 1% alginate (Sigma) and the alginate beads were performed. The chondrogenic medium consisted of DMEM/Ham's F12 (3:1) supplemented with 50 ng/ml ascorbic acid, 1% ITS (insulin-transferrin-sodium selenite media supplement) (Sigma), 5 μg/ml transforming growth factor (TGF)-β3 (Sigma), 20 μM dexamethasone (Sigma), 5 μg/ml amphotericin B and 100 μg/ml penicillin/streptomycin. Cells were incubated for 3 weeks and chondrogenesis was confirmed by the expression of collagen II. In the three aforementioned differentiation protocols, ASCs from both populations cultured in standard medium served as a control.

To analyse the ASCs surface markers, cluster of differentiation (CD)34 (20 μl/test), CD44 (20 μl/test), CD45 (10 μl/test) and CD90 (10 μl/test; BD Pharmingen) were used. Results of flow cytometry were analysed with the FACS Canto CII (BD Biosciences).

### Cell surface marker screening analysis by Lyoplate technology

The BD Lyoplate™ Human Cell Surface Marker Screening Panel, which contains 242 purified monoclonal antibodies to cell surface markers (BD Bioscience), was used to characterize cultured ASCs. The panel also contains isotype controls for assessing isotype-specific background. The assay was processed according to protocol provided by the manufacturer. Briefly, ASCs (passage 2), obtained due to both liposuction procedures, were detached from culture surface by Accutase™ (BD Bioscience). The single cell suspension of live cells was prepared. The cells were washed several times with 1× PBS and centrifuged. More than 3 × 10^8^ of cells was resuspended in BD Pharmingen Stain Buffer supplemented with 5 mM EDTA (Sigma). Then, 2.5 × 10^5^ cells were resuspended in 100 μl aforementioned staining buffer per 96-wells and stained in each well with the specific primary antibody (20 μl) for 30 min on ice. Then, the cells were washed and stained with the secondary antibody (100 μl) for 25 min on ice in the dark. Finally, after washing and centrifuging, the cells were resuspended in 150 μl in BD Pharmingen Stain Buffer/EDTA and measured (3 × 10^4^/well) in FACSCanto II cytometer (BD Bioscences). Data are presented as a heat map generated by BD FACSDiva™ software and Excel 2007. Each experiment was repeated five times.

### Statistical analysis

Data were presented as mean ± S.D. and statistically significant difference was defined as *P*-value<0.005. Statistical analysis was performed using a two-tailed Student's *t*test with SPSS, version 12.0.

## RESULTS

ASCs purification has been performed by prolonged culture of SVF, relying on the ability of ASCs to outcompete other cell populations under the culture conditions over time. Non-adherent cells were washed away and attached cells were recognized as potentially ASCs. These cells were characterized by their immunophenotype in the undifferentiated state and by their differentiation potential towards the adipogenic, osteogenic and chondrogenic lineages in the presence of lineage-specific induction factors. Due to obtained results the cells were recognized as ASCs.

Changes in morphology of ASCs, from the spindle shape cells to round, started appearing after 8 days in differentiation medium toward adipocytes. The presence of fat droplets in the cells' cytoplasm was observed after 2 weeks of culture in both populations (ASCs–MAL and ASCs–UAL) comparing with the control (Figures 1A–1D). The induction of osteogenesis also caused the change in ASCs morphology in the first week of culture in the presence of differentiation factors. The stained calcium deposits confirmed the osteogenesis after 2 weeks in both populations of ASCs in comparison with the control (Figure 2A–2D). The chondrogenic potential of ASCs was evaluated by *in vitro* culture of these cells in micropellet in differentiating medium. After 3 weeks of differentiation, they're showed the morphology similar to hyaline cartilage. Differentiated cells revealed strong expression of collagen type II compared with the control (Figure 3A–3D).

**Figure 1:**
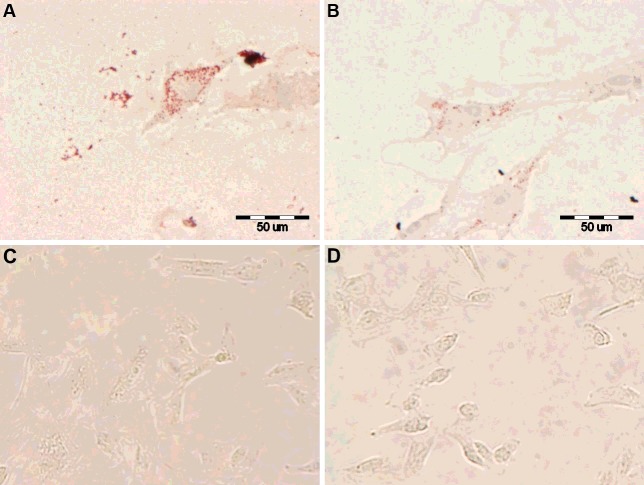
Adipogenic differentiation analysis. ASCs adipogenesis was confirmed, in comparison with the control, through histology, using an oil red stain (**A** and **C**) ASCs obtained due to mechanically assisted liposuction, (**B** and **D**) ASCs obtained due to ultrasound assisted liposuction.

**Figure 2:**
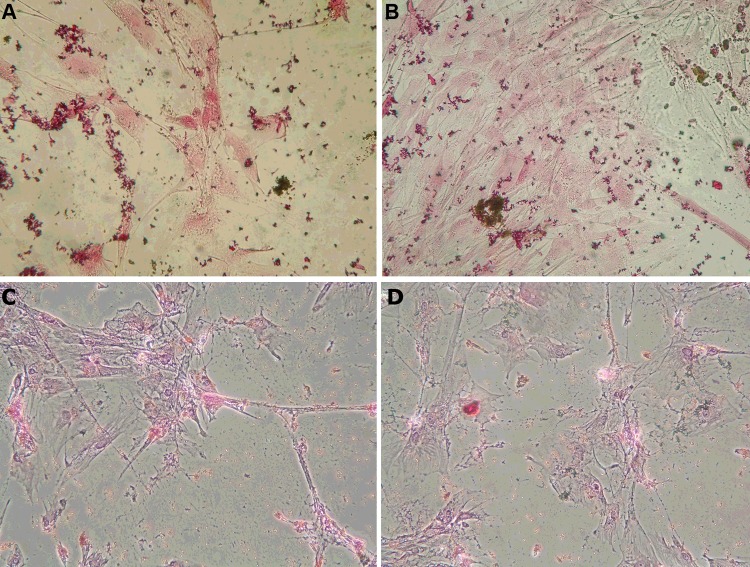
Osteogenic differentiation analysis. To confirm osteogenesis, cells were stained for ECM calcification using von Kossa stain. ASCs cultured in standard culture medium served as a control (**A** and **C**) ASCs obtained due to mechanically assisted liposuction, (**B** and **D**) ASCs obtained due to ultrasound assisted liposuction.

**Figure 3:**
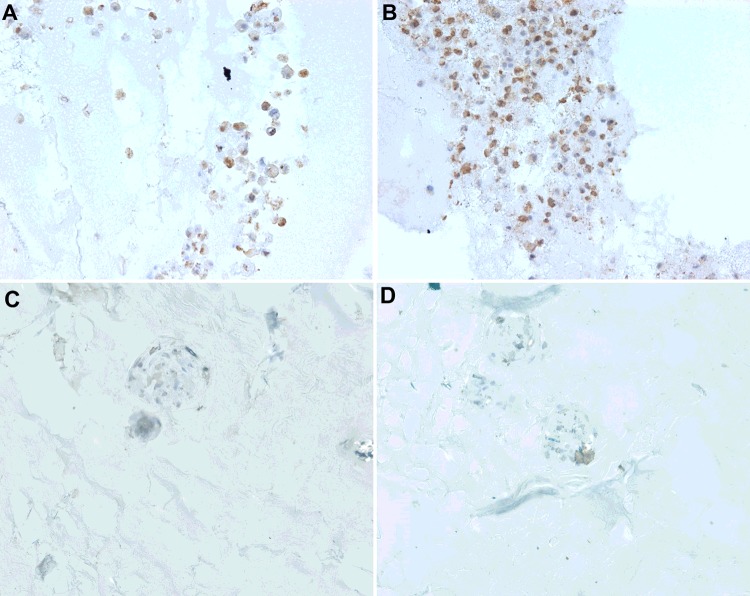
Chondrogenic differentiation analysis. ASCs chondrogenesis was confirmed by expression of collagen II in comparison with the control (**A** and **C**) ASCs obtained due to mechanically assisted liposuction, (**B** and **D**) ASCs obtained due to ultrasound assisted liposuction.

To confirm the mesenchymal stem cells phenotype, the analysis of CD34, CD44, CD45 and CD90 markers was performed in ASCs–MAL and ASCs–UAL after 2 passages (Table 1). Both populations of ASCs were characterized by strong expression of CD90 and CD44 that are specific for the mesenchymal stem cells. Expression of the CD34, marker for haematopoietic progenitor cells, which distinguish the ASCs from other mesenchymal stem cells, was 56.5±0.3 for ASCs-MAL and 75.5±0.1 for ASCs-UAL (Table 1). We observed that this expression decreased in following passages (result not shown). The study also showed no significant contamination with haematopoietic cells due to low expression of CD45 (Table 1).

**Table 1:** Comparison of selected surface markers expression (%) on ASCs, after second passage, obtained due to MAL and UAL

Population of ASCs	CD90*	CD44	CD34*	CD45
ASCs–MAL	88.6±5.0	94.9±1.3	56.5±0.3	6.6±2.8
ASCs–UAL	97.3±0.1	97.9±0.4	75.5±0.1	6.3±2.4

*Statistical significance *P*<0.005

After confirmation of the mesenchymal nature of isolated cells, both populations of ASCs from second passage were evaluated by the Lyoplate measurements for their expression of 242 markers. Our study is the first comprehensive phenotype characterization of cultured ASCs isolated due to mechanical and ultrasound-assisted liposuction. Moreover, for the first time in such kind of analysis the cultured cells were not mixed among different passages. Both cultures of ASCs, used for the Lyoplate assay, were performed in passage 2, which gives 15 days of culture time.

All data collection and automatic evaluation was carried out with the use of a FACSCanto II flow cytometry. The background fluorescence has been set manually for each plate and compared with the isotype controls (both negative). Antigen expression was evaluated by marker and analysis of percentage positive cells. The results have been shown as a heat map (Figures 4 and 5). Characteristic markers for mesenchymal stem cells were expressed in both analysed cells populations: CD13, CD29, CD73, CD90, CD105, CD166. The only statistical difference was noticed according to CD166 marker, whose expression was higher in ASCs-UAL cells. However, the main mesenchymal markers were quantified on the similar level in both cell populations. Analysis also revealed the lack of uncharacteristic markers such as CD11b, CD14, CD19, CD31, CD45 and HLA-DR (human leukocyte antigen-DR) (Table 2). The statistical difference was observed due to three markers: CD31, CD45 and HLA-DR.

**Figure 4:**
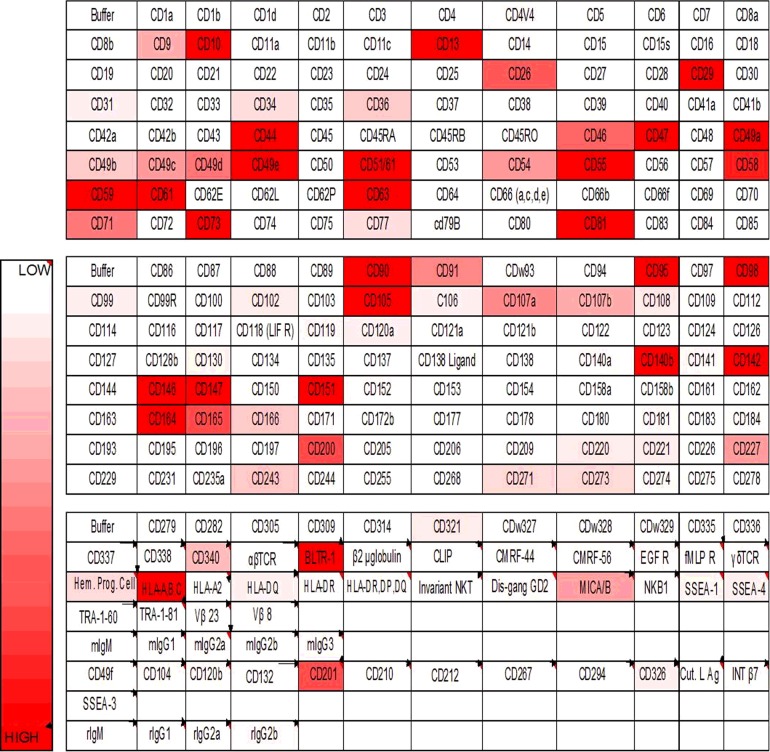
The heat map of ASCs-MAL surface marker expression by Lyoplate assay

**Figure 5:**
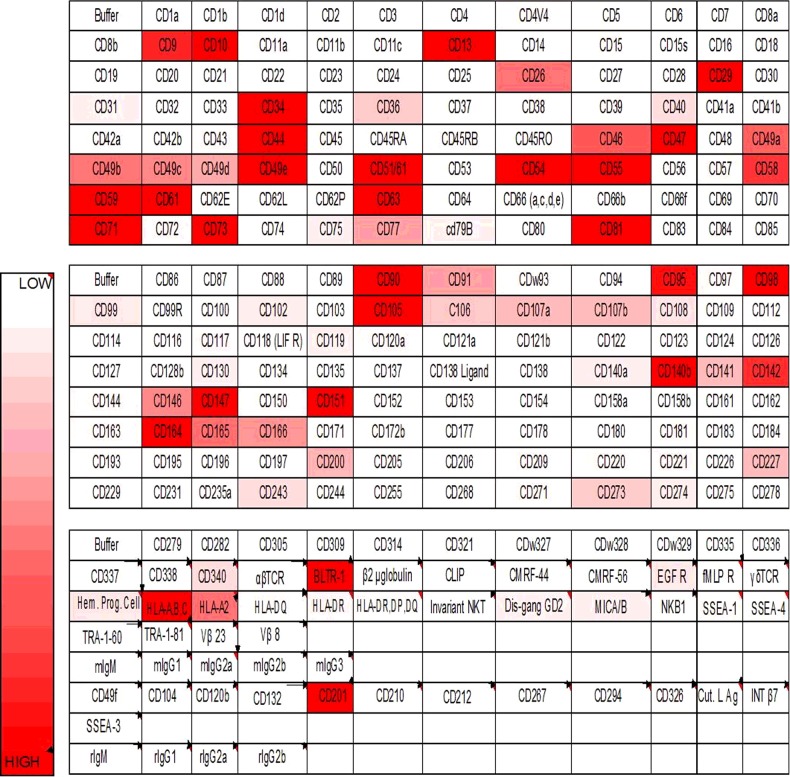
The heat map of ASCs-UAL surface marker expression by Lyoplate assay

**Table 2:** Expression of selected markers (%) characteristic for the mesenchymal stem cells phenotype (MSCs)

Marker	ASCs–MAL	ASCs–UAL
Expression of characteristic markers for MSCs		
CD13	92.7±10.3	96.1±1
CD29	94.3±5.3	96±1.8
CD73	94.1±2.5	95.4±1.6
CD90	98.5±0.9	95.7±1.8
CD105	98.1±0.2	97±1.3
CD166*	37.1±1.1	61±2.7
Expression of uncharacteristic markers for MSCs		
CD11b	1.1±0.6	1±0.4
CD14	1.5±0.7	1.5±0.2
CD19	1.8±1.4	0.8±0.2
CD31*	2.4±1.1	6.6±0.8
CD45*	2.2±1	5.2±0.6
HLA-DR*	1.9±0.4	5.2±0.1

* Statistical significance *P*<0.005

Moreover, both ASCs (isolated due to MAL and UAL) were characterized by high expression of markers specific for ASCs: CD9, CD10, CD34, CD44, CD49d, CD54, CD55, CD59, CD71 and low expression of CD11a, CD11c and CD144 which are described as a non-specific ones (Table 3).

**Table 3:** Expression of selected surface markers (%) characteristic for ASCs

Marker	ASCs–MAL	ASCs–UAL
Expression of characteristic markers for ASCs		
CD9*	38.7±8.9	70.5±5.3
CD10	85.4±6.9	92.7±2.1
CD34*	25.5±4.8	70.2±2.8
CD44	89.7±7.1	94.4±1.2
CD49d	76.4±10.3	68.4±6.8
CD54	78.7±16.3	93.8±2
CD55	96.2±2.3	97.7±0.9
CD59	98.4±1.3	99.4±0.1
CD71	72.4±6.7	87.9±3.5
CD140a*	2±0.1	13.1±0.4
Expression of uncharacteristic markers for ASCs		
CD11a	1.5±0.8	4.4±1.8
CD11c	1.8±1	2.4±0.6
CD106*	13±4.2	38.1±1.8
CD144	1.9±0.3	3.4±1.3

* Statistical significance *P*<0.005

Statistical analysis revealed significant differences in antigen expression of 58 markers of 242 that were studied.

## DISCUSSION

In recent years, many studies have been conducted to characterize the surface markers of ASCs and compared their expression in freshly isolated cells, as well as those maintained in culture in early and late passages [6,8–11]. Unfortunately, all previous attempts to determine the exact phenotype of ASCs and identify specific markers of these cells were unsuccessful [12]. On the basis of researchers conducted so far, in June 2013, the International Society of Cell Therapy (ISCT) and the International Federation for Adipose Therapeutics and Science (IFATS) made a statement concerning the minimum criteria defining a fraction of SVF cells and ASCs. These criteria are similar to those defining the MSCs; however, they also have to distinguish the ASCs population [13]. According to aforementioned statement, ASCs do not show the expression of haematopoietic markers, such as CD11b and CD45 (<2%) and show a high expression of CD13, CD73 and CD90 (>90%). In order to distinguish the ASCs from the bone marrow MSCs it is proposed to use two markers in analysis, i.e. CD36 (GPIIIb, platelet glycoprotein IIIb) and CD106 (VCAM-1, vascular cell adhesion molecule-1). ASCs in contrast with MSCs do not show the expression of CD106, but are CD36 positive [14]. Additional studies evaluating the differentiation potential of ASCs have complimented this profile, suggesting that these cells have a specific expression of CD34 marker and the absence of CD31/CD45 [15]. However, expression of CD34 depends on the culture condition. Generally, CD34 is present during the early period of culture and then decreases with consequent cell divisions [16,17]. It is proposed that the basic characteristics of ASCs included at least two positive and two negative expressions of markers during one analysis. Using additional markers the characteristics of ASCs is strengthen. However, expression of some markers may be different depending on the donor and the passage of examined cells.

ASCs, in the first days of culture, do not show equal expression of characteristic markers [18,19]. Phenotype of these cells can change during *in vitro* culture. In literature differences in expression of some antigens can be found. It may be caused by the investigation of different passages of cultured cells. ASCs in the second or third passage are morphologically homogeneous population and express CD29, CD44, CD73, CD90, CD105 and CD166 and do not show expression of CD11b, Cd14, CD31 and HLA-DR [18].

Based on aforementioned literature data, we selected cells only from the second passage. It is well described that different factors such as liposuction sides, BMI or age can have influence on the phenotype of ASCs. That is why we also distinguish patients with similar parameters (e.g.: age, BMI) to avoid additional variables, which can affect the analysis. Extensive analysis of 242 surface markers revealed the high expression of markers characteristic for MSCs in both ASCs, isolated from the lipoaspirate harvested during MAL and UAL: CD13, CD29, CD73, CD105 and CD166. Both ASCs also show presence of CD90 and CD44, markers typical for stem cells. Analysis revealed the low expression of CD11b, CD14, CD31 and HLA-DR. These results are consistent with other data [20–22]. CD34, which is characteristic for haematopoietic progenitor cells, was noticed in both populations. However, higher expression level was observed in ASCs-UAL (70.2±2.8 compared with 25.5±4.8%). Although, the role of CD34 is well described, its implication in ASCs is still unclear. Some studies suggest that the expression of CD34 indicate cell immaturity. Our additional study revealed the loss of CD34 expression in subsequent passages (result not shown), which can be caused by commitment of cells. It is also well documented that freshly isolated ASCs express different surface markers than ASCs in higher passages [23,24]. Haematopoietic cells did not contaminate analysed ASCs as the expression of CD45 was low in both samples. Expression of analysed markers is similar to the results gained by Baer et al. [23] who used the same technology [18]. However, they do not compare this expression between different methods of adipose tissue collection. Moreover, analysed cells were among 2–4 passages. They showed that 49 markers of the 242 represented high variability between the different donors. It indicates on donor-specific variability in ASCs preparation. In our study, we observed significant differences in antigen expression of 58 markers. However, it is worth to mention that among the characteristic markers for ASCs the significant difference is rather small. It has been shown that different donors, age, BMI and gender or ethnicity influence on cells' number, proliferation and antigens expression [23,25]. It has been also reported that liposuction side and procedure play an important role in the frequency of stem cells [25,26]. There is no data concerning the antigen expression depending on the different liposuction procedure. Our study is the first, which presents such comprehensive markers analysis. We can assume that the applied method of liposuction has no significant impact on the antigen profile in cultured ASCs since there is no important differences in markers characteristic for these cells (according to ISCT and IFATS).

## CONCLUSIONS

In conclusion, our study is the first, which compares the phenotypic pattern according to different methods of adipose tissue collection. It shows for the first time that different methods of sample collection are not a significant factor that can influence the expression of surface markers of ASCs. These data are a step towards standardization of the transplantation procedure of human ASCs for clinical applications.

## Abbreviations

ASC: adipose-derived stem cell
BMI: body mass index
CD: cluster of differentiation
DMEM: Dulbecco's modified Eagle's medium
ECM: extracellular matrix
IFATS: International Federation for Adipose Therapeutics and Science
ISCT: International Society of Cell Therapy
MAL: mechanical-assisted liposuction
SFV: stromal-vascular fraction
UAL: ultrasound-assisted liposuction
